# Site-directed mutation of β-galactosidase from *Aspergillus candidus* to reduce galactose inhibition in lactose hydrolysis

**DOI:** 10.1007/s13205-018-1418-5

**Published:** 2018-10-16

**Authors:** Zhiwei Zhang, Fenghua Zhang, Liya Song, Ning Sun, Weishi Guan, Bo Liu, Jian Tian, Yuhong Zhang, Wei Zhang

**Affiliations:** 1 College of Forestry, Shanxi Agricultural University, Taigu, Shanxi 030801 People’s Republic of China; 2 Biotechnology Research Institute, Chinese Academy of Agricultural Sciences, No. 12 Zhongguancun South Street, Beijing, 100081 People’s Republic of China; 3 Beijing Key Lab of Plant Resource Research and Development, Beijing Technology and Business University, Beijing, 100048 People’s Republic of China

**Keywords:** *Aspergillus candidus*, β-Galactosidase, Galactose inhibition, Lactose hydrolysis, Molecular modification

## Abstract

β-Galactosidase is widely used for hydrolysis of whey lactose. However, galactose inhibition has acted as a major constraint on the catalytic process. Thus, it is sensible to improve upon this defect in β-galactosidase through protein modification. To reduce the galactose inhibition of *Aspergillus candidus* β-galactosidase (LACB), four amino acid positions were selected for mutation based on their molecular bindings with galactose. Four mutant libraries (Tyr96, Asn140, Glu142, and Tyr364) of the LACB were constructed using site-directed mutagenesis. Among all of the mutants, Y364F was superior to the wild-type enzyme. The Y364F mutant has a galactose inhibition constant (*K*_i_) of 282 mM, 15.7-fold greater than that of the wild-type enzyme (*K*_i_ = 18 mM). When 18 mg/ml galactose was added, the activity of the wild-type enzyme fell to 57% of its initial activity, whereas Y364F activity was maintained at over 90% of its initial activity. The wild-type enzyme hydrolyzed 78% of the initial lactose (240 mg/ml) after 48 h, while the Y364F mutant had a hydrolysis rate greater than 90%. The β-galactosidase Y364F mutant with reduced galactose inhibition may have greater potential applications in whey treatment compared to wild-type LACB.

**Electronic supplementary material:**

The online version of this article (10.1007/s13205-018-1418-5) contains supplementary material, which is available to authorized users.

## Introduction

Approximately, 85% of the milk used for manufacturing cheese is discarded as whey (Panesar and Kennedy 2012). Recovery of whole whey solids as ingredients for human or animal food has been a common approach adopted by large industrial processors. Since the main component (70–72%) of whey powder is lactose, direct utilization of whey is impeded by its poor sweetening power, low solubility, and lactose intolerance. Hydrolysis of lactose to monosaccharides, however, significantly increases the options to producing various by-products from whey. For example, hydrolyzed lactose has greater sweetening power and capability to replace saccharose or starch syrup in confectionery and ice-cream industries (Panesar et al. 2006). Hydrolyzed lactose can act as a substrate to produce d-tagatose, an important hexoketose monosaccharide sweetener with health-care functions (Oh 2007), which can greatly increase the additional output of whey in dairy processes.

β-Galactosidase (E.C. 3.2.1.23), also known as lactase, has been suggested for hydrolyzed-lactose milk production and whey hydrolysis (Panesar et al. 2006). However, complete hydrolysis at high lactose concentrations is difficult due to inhibition by galactose and glucose, which can slow the hydrolysis process or even stop the reaction (Park et al. 2010b). Galactose acts as a competitive inhibitor of microbial β-galactosidases by forming galactosyl–enzyme intermediates with β-galactosidase, preventing lactose from entering the active site (Gosling et al. 2010).

The mutation of β-galactosidase from *Caldicellulosiruptor saccharolyticus* can clearly reduce galactose inhibition in lactose hydrolysis (Kim et al. 2011). However, β-galactosidase derived from *Caldicellulosiruptor saccharolyticus* must pass a series of assessment before it can be applied to industrial food practices. The β-galactosidases of commercial interest are isolated mainly from *Kluyveromyces* spp., *Candida kefyr* yeast and the *Aspergillus* spp. fungi (Holsinger and Kligerman 1991; Grosová et al. 2008). *Aspergillus candidus* β-galactosidase (LACB, Uniprot entry: Q8TFE6) has excellent enzymatic properties, including high thermostability, high specific activity, and a wide pH range for enzymatic reactions compared to the commercial enzyme from *Aspergillus oryzae* ATCC 20,423 (Zhang et al. 2002). However, LACB is also seriously inhibited by galactose during whey lactose hydrolysis, and a large amount of galactose is produced during the process. For this reason, it is worth attempting to modify LACB for applications in the whey industry. The crystal structure of *Aspergillus oryzae* β-galactosidase (LACA, Uniprot entry: Q2UCU3) has been determined at a 2.60 Å resolution, and four galactose-binding sites were suggested to exist on the enzyme (Maksimainen et al. 2013). As LACB and LACA share high sequence similarity and both belong to the glycoside hydrolase 35 (GH-35) family, the latter was explored as a template to determine the galactose-binding residues in LACB. In this study, the predicted residues were engineered to reduce galactose inhibition.

## Materials and methods

### Strains, plasmids and media

*Escherichia coli* Trans1-T1 cells (TransGen Biotech, Beijing, China) and *Pichia pastoris* GS115 (Invitrogen, CA, U.S.A) were used as gene cloning and expression hosts, respectively. The *P. pastoris–E. coli* shuttle expression vector pPIC9-*lacb* was previously constructed in our laboratory (Zhang et al. 2002). Media, including minimal dextrose (MD) medium, minimal methanol (MM) medium, and yeast peptone dextrose (YPD) medium, were prepared according to instructions in the *Pichia* expression kit (Invitrogen). Fermentation basal salts (FBS) medium and *Pichia* trace metal (PTM) complied with *Pichia* fermentation guidelines (Invitrogen).

### Construction of the mutation library

The to-be-mutated sites in *Aspergillus candidus* β-galactosidase (LACB) were determined by sequence alignment between LACB and *Aspergillus oryzae* β-galactosidase (LACA). These two enzymes are both in the CAZy (http://www.cazy.org/) GH-35 family with high sequence similarity (99.3%) and a close evolutionary relationship. The galactose-binding sites in the LACA have been previously reported (Maksimainen et al. 2013). Residues Tyr96 (Y96), Asn140 (N140), Glu142 (E142) and Tyr364 (Y364) in LACB that were similar to galactose-binding sites in LACA were chosen for mutation.

Two pairs of primers were used to construct the mutated expression vector from pPIC9-*lacb* for each of the selected sites. For example, for the Y96 site mutation, primer pairs Bgl II F-Y96R and Y96F-Bgl II R were used; two fragments were amplified using pPIC9-*lacb* as a template, one of which carried mutated bases. These two fragments share 20 bp homologous sites at both ends and were recombined into an entire plasmid using CloneEZ recombinant enzyme (Genscript, Nanjing, China) according to instructions. For the N140 site mutation, primer pairs Bgl II F-N140R and N140F-Bgl II R were used, and a similar method was used for the remaining sites. Primers for mutation are listed in Table 1.

**Table 1:** Primers for saturated mutagenesis

Mutant library	Primer name	Primer sequence (5′-3′)^a^
	BglIIF	ACGTGAAATTTATCTCAAGATCTCTGCCTCGCG
	BglIIR	CGCGAGGCAGAGATCTTGAGATAAATTTCACG
Y96	Y96F	ATTGATTGGGCTCTTCTGGAGGGAAAGCC
	Y96R	CCCTCCAGAAGAGCCCAATCAAT**MNN**GAAAGATAC
N140	N140F	GCCGAGGTCTCAGGCGGTGGCTT
	N140R	CCACCGCCTGAGACCTCGGC**MNN**GATG
E142	E142F	GTCTCAGGCGGTGGCTTCCCTGGA
	E142R	AAGCCACCGCCTGAGAC**MNN**GGCATT
Y364	Y364F	GGCTCGCCTATAACTGAAACGCGAAACGTTAC
	Y364R	GTTTCAGTTATAGGCGAGCC**MNN**GTCGTAGG

^a^The underlined words indicate the mutated base. M represents A or C; N represents A, T, G, or C

### Heterologous expression of enzymes

Mutant pPIC9-*lacb* plasmids were transformed into *E. coli* Trans1-T1. Twenty colonies were randomly selected from each library and sequenced to detect the mutant rate of nucleotide sequences. All *E. coli* transformants were collected and used for mixed plasmids extraction. The mixed mutant pPIC9-*lacb* plasmids were transformed into *P. pastoris* GS115. Electroporation of *P. pastoris* with mutated pPIC9-*lacb* was carried out according to the manufacturer’s instructions. Transformants were cultured on MD plates at 28 °C for 72 h and incubated on MM plates with 80 µl of 40 mg/ml X-gal (5-bromo-4-chloro-3-indolyl β-d-galactoside) for another 24 h. Blue plaques were considered positive; these were proliferated in 48-well plates of YPD broth for 48 h. The cells were then harvested by centrifugation at 4000*g* for 5 min and grown in FBS medium for 24 h, followed by a second centrifugation and growth in FBS-inducing medium (20 g glucose/l replaced by 0.5% methanol, v/v) to produce the enzymes of interest. Methanol was supplemented with 0.5% (v/v) every 12 h. For purification, the cultivation course was carried out in 250-ml flasks.

### Purification of enzymes

The culture supernatants were harvested by centrifugation at 4000*g* for 10 min, then condensed and desalted with a 10-kDa ultrafiltration membrane. β-Galactosidase was purified by passing through an anion exchange column CaptoQ (GE Healthcare Life Sciences, CA, USA) and eluted with an eluent solution (20 mM citrate/phosphate buffer containing 1 M NaCl, pH 7.5) in an AKTA purifier chromatographic system (GE Healthcare Life Sciences) according to Nie et al.’s method (Nie et al. 2013).

### Enzyme assay

The reaction was performed at 60 °C for 15 min with citrate/phosphate buffer (0.1 M, pH 5.2) containing 800 µl of 0.25% (w/v) *o*-nitrophenyl-β-d-galactopyranoside (*o*NPG) and 200 µl of enzyme solution. The reaction was terminated by addition of 1 ml 10% (w/v) trichloroacetic acid (TCA) and 2 ml 1 M Na_2_CO_3_, and the absorbance was measured at 420 nm. One unit of hydrolytic activity (U) was defined as the amount of β-galactosidase required to release 1 µmol *o-*nitrophenol per minute under standard conditions (pH 5.2, 60 °C). The remaining activity, which was the activity measured in the presence of 24 mg/ml galactose, was defined as a relative value to the activity in absence of galactose.

The protein concentrations were determined by a Bio-Rad Protein Assay Kit (Bio-Rad, CA, USA) according to its instructions. The ratio of enzyme activity (U/ml) to enzyme concentration (mg/ml) was referred to as the specific activity.

#### Galactose inhibition against β-galactosidase

To analyze the effect of galactose on β-galactosidase activity, reactions catalyzed by wild-type and mutant enzymes were performed in citrate/phosphate buffer (0.1 M, pH 5.2) containing 0.25% *o*NPG with varying concentrations of galactose (from 0 to 90 mg/ml). The galactose inhibition constant (*K*_i_) was analyzed with GraphPad Prism 5.0 software by measuring β-galactosidase activity at various concentrations of galactose (from 0 to 90 mg/ml) and of *o*NPG (in the range of 4 to 20 mM) as substrates.

#### Lactose hydrolysis

Time course hydrolysis of lactose by β-galactosidase was performed in citrate/phosphate buffer (0.1 M, pH 5.2) containing 240 mg/ml lactose, with or without 24 mg/ml galactose, and equal aliquots of enzyme (40 µg/ml) to evaluate the hydrolytic performance of wild-type and mutant enzymes. The reactions were halted by incubation at 100 °C for 10 min, and the lactose content was determined by high-performance liquid chromatography (HPLC, Waters, Boston, MA, USA).

### Protein modeling, molecular docking and dynamics

The structures of the studied enzymes were modeled using the SWISS-MODEL. Molecular docking and dynamics were examined using Discovery Studio 2.55.

## Results

### Determination of possible galactose-binding residues in LACB

The protein sequence of *Aspergillus candidus* β-galactosidase (LACB) shares 99.3% similarity with *Aspergillus oryzae* β-galactosidase (LACA), implying similar tertiary structures and possibly galactose-binding residues between the two. In LACA, galactose forms hydrogen bonds with Tyr96 (Y96), Asn140 (N140), Glu142 (E142), and Tyr364 (Y364) (Maksimainen et al. 2013); LACB has the same residues in the same positions. Thus, Y96, N140, E142, and Y364 are presumably galactose-binding residues in LACB and were selected for mutation.

### Construction and selection of the mutant library

Four single-point-mutant *E. coli* libraries (Y96, N140, E142, and Y364) were constructed using the site-directed saturation mutagenesis method. Each amino acid position was mutated to each of the 19 other amino acids, respectively. The mutant rate of each *E. coli* library was above 85%. After transformation into *P. pastoris*, the positive clones were screened by chromogenic reactions (Supplementary Figure S1). Finally, at least 200 positive transformants of each *P. pastoris* mutant library were selected for further determination.

### Determination of remaining activity and specific activity

**Fig. 1:**
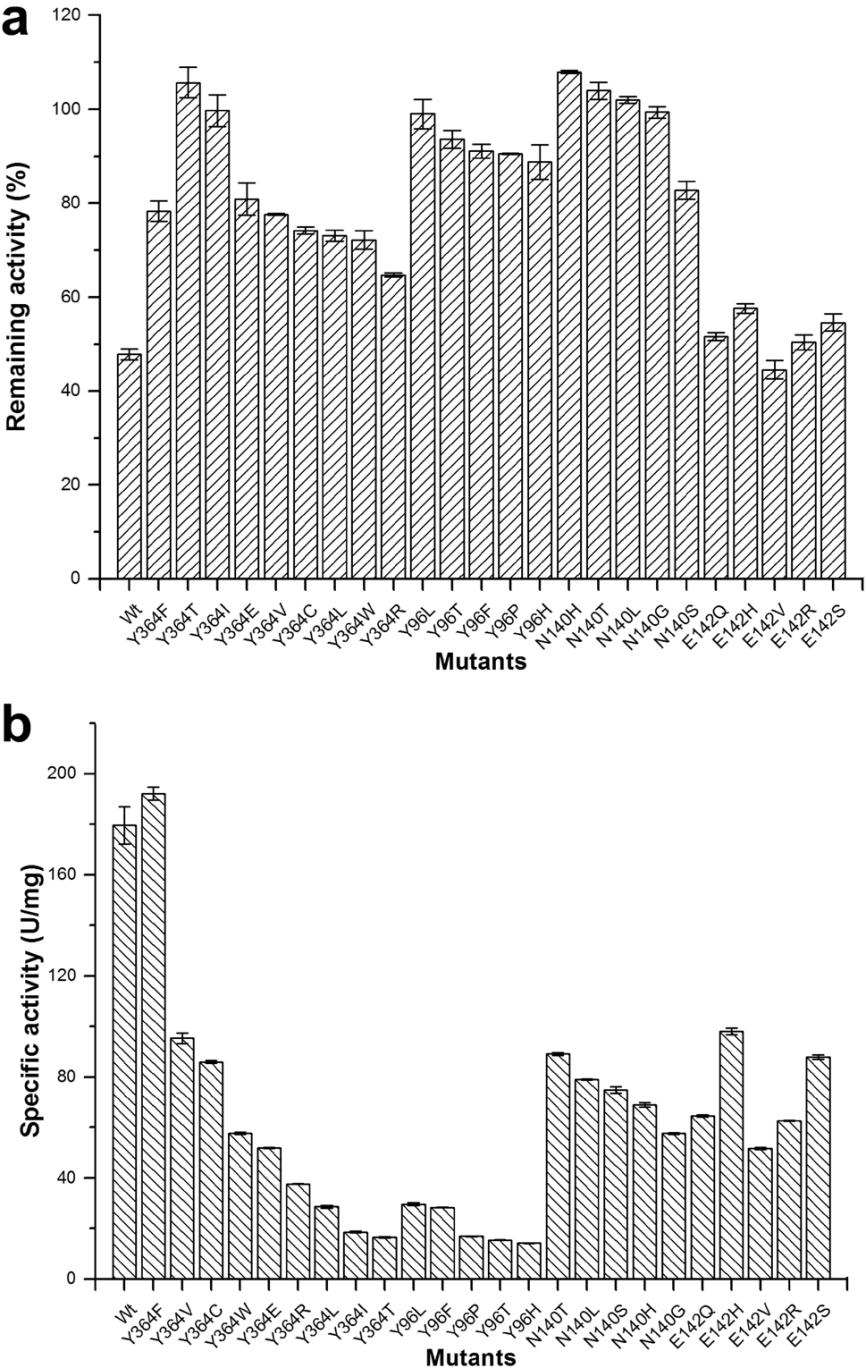
β-Galactosidase remaining activity (**a**) and specific activity (**b**) of the wild-type enzyme (Wt) (*Aspergillus candidus* β-galactosidase, LACB) and mutants. **a** The reactions catalyzed by the wild-type (Wt) and mutant enzymes were performed in citrate/phosphate buffer (0.1 M, pH 5.2) containing 0.25% *o*NPG (*o*-nitrophenyl-β-d-galactopyranoside) with 24 mg/ml galactose. The remaining activity was defined as the relative value of β-galactosidase activity measured in the presence of galactose to the activity measured in the absence of galactose. The respective β-galactosidase activity of each protein (200 µg/ml protein, between 4 and 40 U/ml) measured without galactose was defined as 100%. **b** The protein concentrations were determined by Bio-Rad Protein Assay Kit (Bio-Rad, CA, USA). The ratio of enzyme activity (U/ml) to enzyme concentration (mg/ml) is referred to as the specific activity. Data represent the means of three experiments, and error bars represent the standard deviation

Except for the E142 site, mutant enzymes for the Y96, N140, and Y364 sites displayed tremendous declines in galactose inhibition with high remaining activities. The wild-type enzyme exhibited 48% remaining activity relative to the activity observed in the absence of galactose, whereas the activity of the Y364F mutant was 78%. The activities of Y96L, N140H, and Y364T remained at nearly 100% (Fig. 1a).

For most of the mutant enzymes at the Y96, N140, E142, and Y364 sites, the specific activities were much lower than those of the wild-type enzyme (Fig. 1b). The specific activity of Y364F was close to that of the wild-type enzyme. The activities of the other mutants were lower: Y364C had an activity of 47.9% of the wild-type’s specific activity, and Y96L had only 16.5%. Accounting for specific and remaining activities, only Y364F displayed superior enzymatic quality over the wild-type enzyme. In summary, the specific activity of Y364F was near that of the wild-type enzyme, whereas the remaining activity improved by 30% compared to the wild type. Therefore, more detailed analyses were performed on the wild-type enzyme and Y364F mutant.

### Galactose inhibition of wild-type enzyme and Y364F mutant

**Fig. 2:**
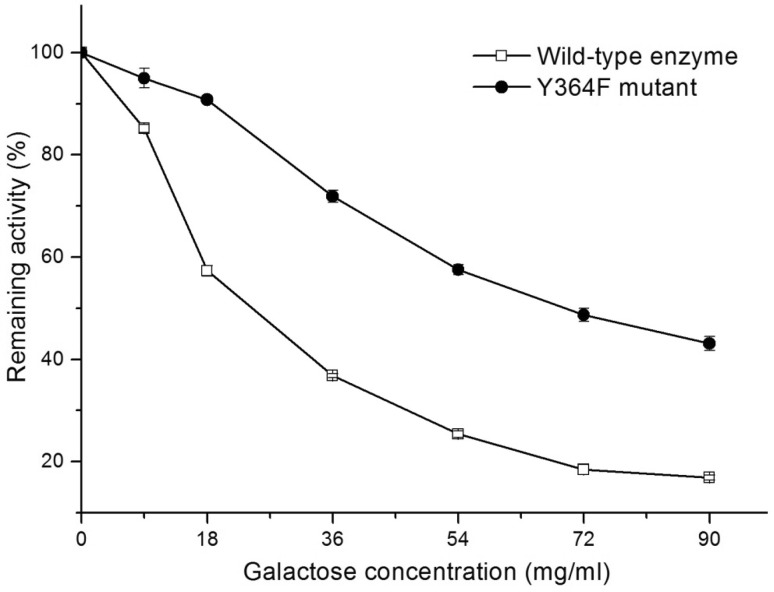
Effects of galactose on wild-type enzyme (square) and Y364F mutant (solid circle). Reactions catalyzed by wild-type and mutant enzymes were performed in citrate/phosphate buffer (0.1 M, pH 5.2) containing 0.25% *o*NPG with varying concentrations of galactose (from 0 to 90 mg/ml). The remaining activity was defined as the relative value of β-galactosidase activity measured with galactose to the maximum activity measured without galactose. The respective β-galactosidase activity of each protein (200 µg/ml protein, 36.2 U/ml for wild-type enzyme and 38.4 U/ml for Y364F mutant) measured without galactose was defined as 100%. Data represent the means of three experiments, and error bars represent the standard deviation

The activity of the wild-type enzyme declines sharply with increased galactose concentration, whereas Y364F maintains higher activity in the presence of galactose (Fig. 2). When the galactose concentration reached 18 mg/ml, the activity of the wild-type enzyme fell to 57% of its initial activity, whereas Y364F activity was maintained at over 90% of its initial activity. This suggests that Y364F is less sensitive to galactose inhibition.

The two enzymes were purified, and their galactose inhibition constants (*K*_i_) were determined. The *K*_i_ of Y364F is 282 mM, about 15.7 times greater than that of the wild-type enzyme (*K*_i_ = 18 mM), which indicates a significant reduction in galactose inhibition in the Y364F mutant. Compared with the reported *K*_i_ of different β-galactosidases (Table 2), Y364F has the highest *K*_i_, implying that it is the least sensitive to galactose.

**Table 2:** Comparison of *K*_i_ values of β-galactosidase with galactose inhibition

Enzyme source	Substrate	*K*_i_ (mM)	References
*A. candidus* (wild-type LACB)	*o*NPG	18^a^	This study
*A. candidus* (Y364F)	*o*NPG	282^a^	This study
*A. niger van Tiegh*	*o*NPG	0.76	Hu et al. (2010)
*Geobacillus stearothermophilus*	*o*NPG	86	Dong et al. (2013)
*A. oryzae*	Lactose	15	Shukla and Chaplin (1993)
*Thermus* sp. T2	*o*NPG	3	Pessela et al. (2003)
*Caldicellulosiruptor saccharolyticus*	*p*NPG	12	Park et al. (2010a)
*Kluyveromyces lactis*	*o*NPG	45	Mateo et al. (2004)
*Lactobacillus reuteri*	*o*NPG	115	Nguyen et al. (2006)
Unculturable microbes	*o*NPG	238	Zhang et al. (2013)

*oNPG o*-nitrophenyl-β-d-galactopyranoside, *pNPG p*-nitrophenyl-β-d-galactopyranoside

^a^The galactose inhibition constant (*K*_i_) was analyzed with GraphPad Prism 5.0 by measuring the β-galactosidase activity using various concentrations of galactose (from 0 to 90 mg/ml) and *o*NPG (in the range of 4 to 20 mM) as substrates

### Lactose hydrolysis

**Fig. 3:**
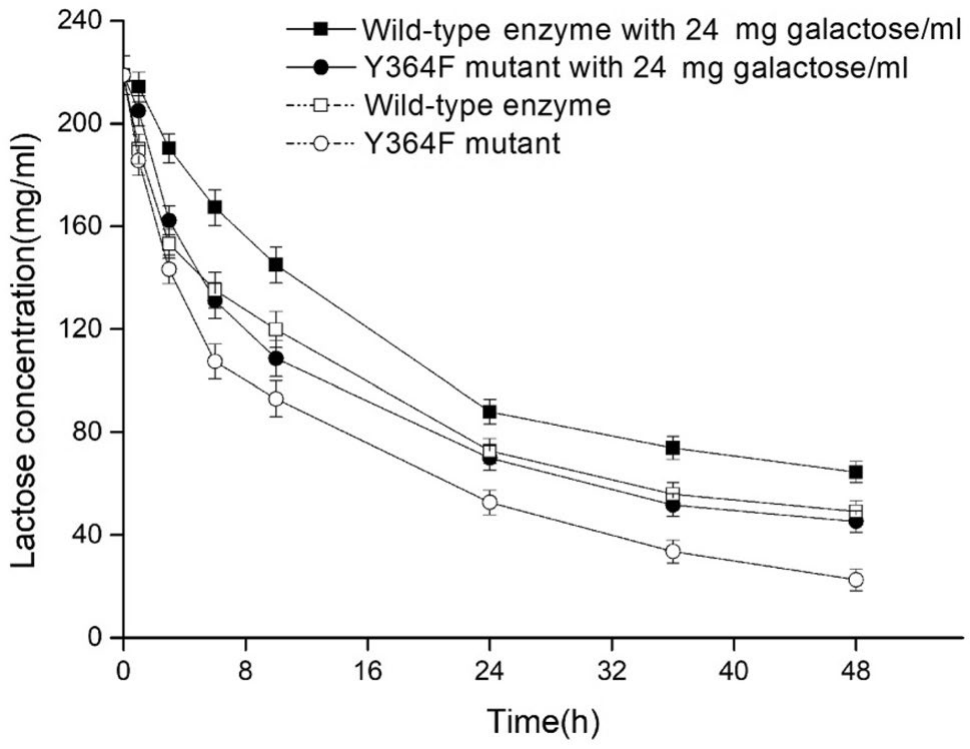
Time course hydrolysis of lactose by wild-type enzyme and Y364F mutant. The reactions were performed at 50 °C for 0, 1 h, 3 h, 6 h, 10 h, 24 h, 36 h, and 48 h in 0.1 M citrate/phosphate buffer (pH 5.2) containing 240 mg /ml lactose, with or without the addition of 24 mg/ml galactose, and equal aliquots of enzyme (40 µg/ml). The reactions were halted by incubation at 100 °C for 10 min, and the lactose concentration was determined by high-performance liquid chromatography (HPLC, Waters, Boston, MA, USA)

Y364F has an advantage over the wild-type enzyme in lactose hydrolysis, as it has a higher hydrolytic rate. When extra galactose was absent, the wild-type enzyme hydrolyzed 78% of the initial lactose, whereas the Y364F mutant hydrolyzed more than 90% after 48 h. When extra galactose was added, the hydrolytic rate of both enzymes fell, but the rate for Y364F remained higher (Fig. 3).

## Discussion

**Fig. 4:**
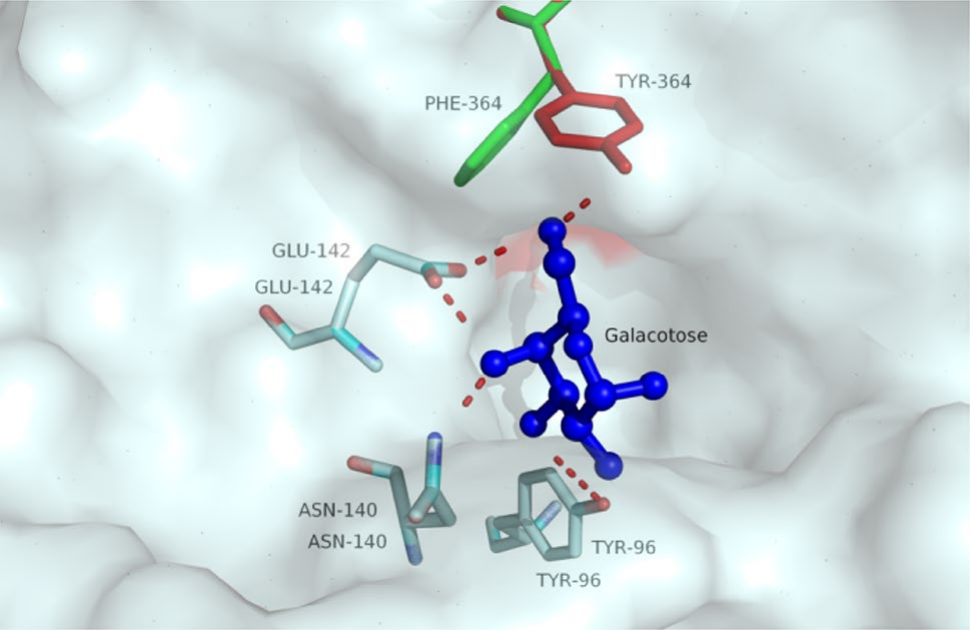
Superimposition between the structures of the wild-type enzyme and Y364F mutant. The red stick model indicates Y364 in the wild-type enzyme. Green indicates F364 in the Y364F mutant. Blue indicates galactose, and the red dash indicates the predicted hydrogen bond

The structure of *Aspergillus candidus* β-galactosidase (LACB) was modeled using *Aspergillus oryzae* β-galactosidase (LACA) as a template. Galactose docking and molecular dynamics were examined using Discovery Studio 2.55. Based on the structure of LACA, one galactose molecule was sighted in the active area of LACB (Fig. 4), forming hydrogen bonds to Tyr96, Asn140, Glu142, and Tyr364 (Maksimainen et al. 2013). It can be inferred that these amino acid sites also have molecular interactions with the substrate lactose. When these amino acid sites mutate, their ability to bind galactose is reduced, so galactose inhibition is weakened, but their ability to bind to lactose substrate may also change. As a result, the specific activity of most mutants fell sharply, as shown in Fig. 1b, and they may not be suitable for lactose hydrolysis. As a special case, Tyr (Y364) is present at the end of the active site and has a hydrogen bond with it, whereas Phe (F364) lacks a hydroxyl group and does not form hydrogen bonds with galactose (Fig. 4), reducing the contact strength between galactose and the active site and causing galactose to be more readily released or galactose competitiveness to decrease. Because of the structural similarity between Phe (F364) and Tyr (Y364), there are no significant changes in the general conformation of LACB; therefore, enzyme activity was retained.

As β-galactosidase plays a major role in converting lactose to glucose and galactose, selection for or by enzyme engineering to attain high-profile β-galactosidase is a popular topic in current enzyme studies. Mutation of galactose-binding sites could be an effective strategy for reducing galactose inhibition. In this study, there is only one difference in hydroxyl groups between the most effective mutant Y364F and wild-type LACB, but this difference has an obvious effect on the enzyme catalysis. An in-depth understanding of protein structure suggests that even slight changes in key amino acids may affect protein properties. This mutation strategy will greatly reduce the workload of screening mutants compared to directed evolution. For other studies, Dong et al. (Dong et al. 2013) identified the galactose-binding sites in thermostable β-galactosidase from *Geobacillus stearothermophilus* through homology modeling and molecular dynamics simulation and through mutating the predicted galactose-binding residues and obtaining a mutant, F341T, that can completely hydrolyze lactose. Kim et al. (Kim et al. 2011) modified ten predicted galactose-binding residues in lactase from *Caldicellulosiruptor saccharolyticus* and finally obtained a mutant, F349S, with a *K*_i_ of 160 mM, which was 13-fold greater that of the wild-type enzyme. However, β-galactosidase derived from *Caldicellulosiruptor saccharolyticus* must pass a series of assessments before it can be applied in industrial food practices. Commercial β-galactosidase enzymes are mainly derived from *A. oryzae* and *Kluyveromyces lactis. A. oryzae* β-galactosidase has a galactose inhibition constant (*K*_i_) of 15, and *K. lactis* β-galactosidase has a *K*_i_ of 45, which is far lesser than that of the Y364F mutant in this study (Table 2). This result indicates that the Y364F mutant has better galactose resistance than commercial β-galactosidase.

In this paper, the mutant Y364F from *Aspergillus candidus*, a commercial species in the fungi genus *Aspergillus*, showed reduced galactose inhibition with a *K*_i_ of 282 mM. Y364F performed much better than the wild-type enzyme and commercial β-galactosidase, thus the mutant may have greater potential for lactose hydrolysis.

## Conclusion

To reduce galactose inhibition in β-galactosidase LACB for industrial purposes, four mutant libraries of LACB were constructed using site-directed mutagenesis. Among all of the mutants, one positive mutant Y364F that displays lower galactose inhibition and higher lactose hydrolytic efficiency was obtained. The Y364F mutant has a galactose inhibition constant (*K*_i_) of 282 mM, 15.7-fold greater than that of the wild-type enzyme (*K*_i_ = 18 mM). The wild-type enzyme hydrolyzed 78% of the initial lactose (240 mg/ml) after 48 h, while the hydrolysis rate of the Y364F mutant was over 90% at the same time. The Y364F mutant of β-galactosidase LACB has good potential in whey lactose hydrolysis.

## Electronic supplementary material

Below is the link to the electronic supplementary material.

Supplementary material 1 (DOCX 2206 KB)
