# Supplementary material for: Site-directed mutation of β-galactosidase from Aspergillus candidus to reduce galactose inhibition in lactose hydrolysis
Source: 3 Biotech. 2018 Oct 16;8(11):452. doi: 10.1007/s13205-018-1418-5 (PMC6191392; doi:10.1007/s13205-018-1418-5)

**Supplementary figure captions**

**Supplementary Figure S1** Chromogenic reactions on the plate to screen *P. pastoris* positive clones. Transformants were cultured on MM plates with 80 μl of 40 mg/ml X-gal (5-Bromo-4-chloro-3-indolyl β-D-galactoside) for 24 h. Blue plaques were considered as positive clones.

**Fig. S1**


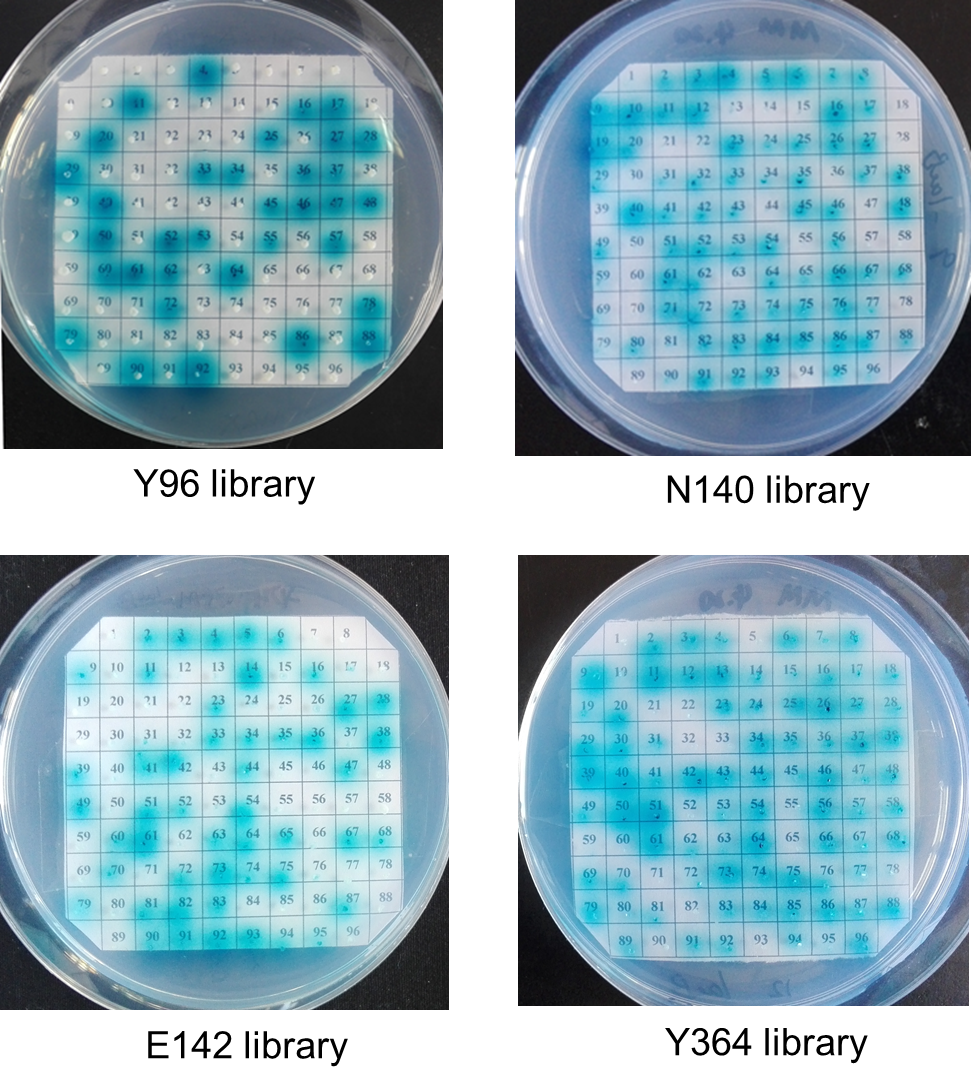

Supplement: Supplementary file 1 — Supplementary material 1 (DOCX 2206 KB) [file 13205_2018_1418_MOESM1_ESM.docx]
